# Acetone Ingestion Resulting in Cardiac Arrest and Death

**DOI:** 10.7759/cureus.18466

**Published:** 2021-10-03

**Authors:** Chukwuemeka Umeh, Rakesh C Gupta, Rahul Gupta, Harpreet Kaur, Shadi Kazourra, Stella Maguwudze, Adrian Torbela, Shipra Saigal

**Affiliations:** 1 Internal Medicine, Hemet Global Medical Center, Hemet, USA; 2 Pulmonary and Critical Care Medicine, Hemet Global Medical Center, Hemet, USA

**Keywords:** nail polish cleaner ingestion, death, respiratory arrest, cardiac arrest, acetone

## Abstract

Acetone is a chemical found naturally in the environment, and acetone poisoning can occur through contact with household products that contain acetone, including nail polish removers, paint removers, some polishes, as well as certain detergents and cleaners. Acetone toxicity affects almost all body systems, including the nervous, respiratory, cardiovascular, and endocrine systems. The incidence of life-threatening medical problems from acetone exposure is very rare. However, in this report, we present a case of acetone toxicity resulting in death. Our patient presented with cardiopulmonary arrest, hypotension, tachycardia, hyperglycemia, acute kidney injury (AKI), thrombocytopenia, elevated liver enzymes, and extensive esophageal and gastric erosion with associated upper gastrointestinal bleeding.

## Introduction

Acetone is a colorless chemical with a distinct taste and smell, and it is found naturally in the environment and produced by industries. Low levels of acetone are usually present in the body from body fat breakdown. Additionally, people are exposed to small amounts of acetone from drinking water, food, or breathing the air that contains acetone. Exposure to acetone can also occur through contact with household products that contain acetone, including nail polish removers, paint removers, some polishes, and certain detergents and cleaners. Acetone exposure also occurs through exposure to isopropyl alcohol since isopropyl alcohol is converted to acetone in the body [1,2].

Several cases of acetone poisoning and accidental human exposure to acetone have been reported, and acetone has been shown to affect almost all body systems, including the nervous, respiratory, cardiovascular, and endocrine systems, among others [1]. However, the incidence of life-threatening medical problems from acetone exposure is very rare, and very few human deaths attributed to acetone exposure have been reported. In a review of 1,553 incidents of human exposure to acetone from the 2019 Annual Report of the American Association of Poison Control Centers National Data Collection System, only one fatality was reported [3]. In some of the fatal cases related to acetone, co-exposure with other chemicals may have occurred [1,3]. Animal studies have shown that exposure to very high acetone concentrations is required to cause death [1]. We present a case of cardiopulmonary arrest and death from acetone ingestion and discuss the clinical presentation and side effects.

## Case presentation

A 41-year-old female patient with underlying chronic obstructive pulmonary disease (COPD) was brought in to evaluate cardiopulmonary arrest after ingesting acetone. Per emergency medical services (EMS), the patient had swallowed two-thirds of a bottle of acetone, and EMS had found drug paraphernalia on site. EMS had found the patient in asystole and started chest compressions, and had administered epinephrine. There had been a return of spontaneous circulation following chest compressions, and the patient had been intubated in the field, and an orogastric tube had been placed. On arrival at the emergency department (ED), the patient's blood pressure was 55/43 mmHg, heart rate was 117 beats per minute, temperature was 98.4 °F, and respiratory rate was 22 breaths per minute. The orogastric tube had bright red blood, pupils were unreactive, and the patient had a Glasgow Coma Scale (GCS) score of 3. Laboratory results showed normal white blood cell count, hemoglobin of 11.8 g/dl with mean corpuscular volume (MCV) of 118 fl, and low platelet count of 69,000/ml. Potassium was low at 3.1 meq/l, and magnesium was low at 1.2 mg/dl (Table 1). Blood PH was 7.24, and serum osmolality was elevated at 305, with both anion and osmolar gaps elevated at 18 and 20, respectively. Aspartate aminotransferase (AST) was elevated at 158 iU/l, alanine transaminase (ALT) was normal, and alkaline phosphatase was elevated at 129 iU/l (Table 1). The patient's blood-alcohol level was elevated at 59 iU/l, and the salicylate and acetaminophen levels were normal. The urine drug screen was positive for amphetamine and cannabinoid. CT brain showed no intracranial hemorrhage, infarct, mass effect, or midline shift.

**Table 1:** Laboratory test values on hospital admission

Laboratory test	Values
White blood cells	8,100/ml
Hemoglobin	11.8 g/dl
Mean corpuscular volume (MCV)	111 fL
Platelet count	69,000/mL
Sodium	136 meq/L
Potassium	3.1 meq/L
Chloride	101 meq/L
Magnesium	1.2 mg/dl
Blood urea nitrogen (BUN)	14 mg/dL
Creatinine	1.17 mg/dL
Aspartate aminotransferase (AST)	158 iU/L
Alanine transaminase (ALT)	41 iU/L
Alkaline phosphatase	129 iU/L

**Figure 1:**
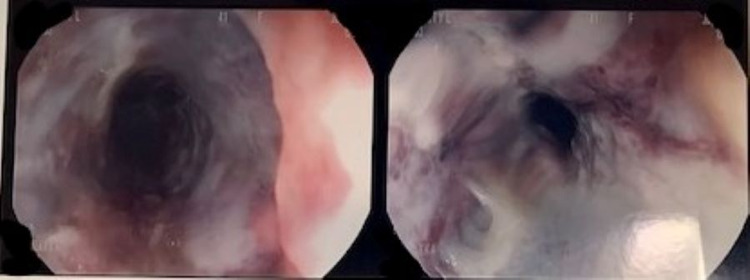
Ulcerated proximal esophagus after acetone ingestion

**Figure 2:**
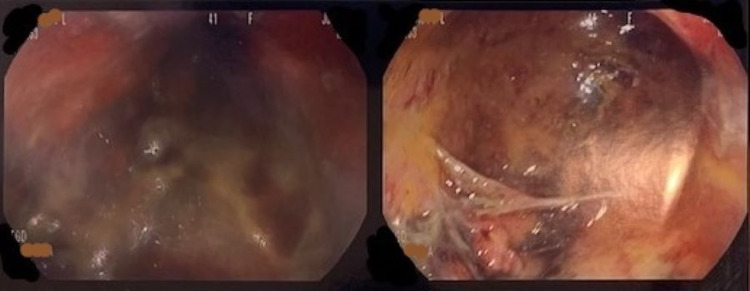
Ulcerated and necrotic stomach after acetone ingestion

In the ED, the patient was started on three vasopressors, IV fluids, and antibiotics. ED physician contacted poison control, and they recommended symptomatic management of the patient. The next day, the patient was hyperglycemic with a blood sugar of 614 mg/dl; anion gap was normal at 8, and hemoglobin A1c was 5.7. The hyperglycemia was attributed to acetone metabolism, and the patient was started on an insulin drip, which was discontinued within 48 hours after blood sugar levels normalized. Creatinine trended up from day one of admission and peaked at 2.08 mg/dl on day three and returned to normal on day five of admission. AST trended down and normalized on day eight of admission. Platelets also trended up and normalized on day five of admission. The patient continued to bleed through the orogastric tube. CT abdomen showed moderate to marked diffuse gastric wall thickening with edema consistent with gastritis secondary to foreign substance ingestion. Esophagogastroduodenoscopy (EGD) on day five of admission showed ulcerated and necrotic esophagus and stomach (Figures 1, 2). The patient's GCS score remained at 3. Electroencephalogram (EEG) showed no cortical activity, but the patient had some brainstem function with some spontaneous breathing. The patient was extubated on day 15 of admission as per the family's wishes, and she died on day 19 of admission.

## Discussion

Acetone metabolism

Acetone is rapidly absorbed from the gastrointestinal tract and the lungs and through the skin [4,5]. After inhalation, acetone absorption increases with increased concentration and duration of exposure, while absorption after oral ingestion appears to increase when consumed on an empty stomach [1,6]. Acetone is widely distributed in tissues and organs in the body because of its high water solubility [2]. The metabolism of acetone is independent of the route of absorption and occurs primarily in the liver. Acetone is mainly excreted through the lung and a little through the urine irrespective of the route of exposure, and elimination is usually completed within 48-72 hours after exposure.

Toxic effects of acetone

Central Nervous System

Acetone exposure has been associated with the central nervous system (CNS) depression, confusion, coma, ataxia, coordination impairment, dizziness, weakness, headache, irritability, stupor, seizure, sleep disturbance, memory difficulty, and difficulty in speaking [7-10].

Gastrointestinal System

Inhalational and dermal exposure to acetone has been associated with nausea, abdominal pain, loss of appetite, vomiting, and gastrointestinal hemorrhage, possibly due to repeated vomiting [8-12]. Erosions of the soft palate and esophagus have been reported with acetone ingestion [7]. Our patient had extensive esophageal and gastric erosion with associated upper gastrointestinal bleeding from acetone ingestion.

Respiratory System

Inhaled acetone irritates the nose, throat, trachea, and lungs, which is likely due to the local action of acetone on the respiratory tract [13-15]. Bronchial edema has also been reported with acetone inhalation [16]. In addition, respiratory distress has been reported following oral exposure to acetone, which may be attributed to CNS depression from acetone or the lung's role in excreting orally ingested acetone [17].

Cardiovascular System

Different cardiovascular effects have been reported with acetone exposure, including hypotension, hypertension, sinus tachycardia, and cardiovascular collapse [17-19]. Our patient presented with cardiac arrest, hypotension, and tachycardia. However, she also had high alcohol levels in her blood, and her urine drug screen was positive for amphetamine and cannabinoid. Therefore, we cannot ascertain if the cardiovascular effects of acetone in our patient were only due to acetone or due to the combined effect of acetone, alcohol, amphetamine, and cannabinoid.

Hematological System

Increased white blood cells (leukocytosis) have been reported in humans exposed to acetone [17,20]. Our patient presented with a normal white blood cell count but had low platelets. The platelet level normalized within five days. She also presented with persistently elevated MCV, which could have resulted from her alcohol use.

Renal System

Acute kidney injury (AKI) has been reported in humans exposed to acetone [16,21]. Polyuria has also been reported with acetone poisoning [1], which might be secondary to hyperglycemia seen in acetone poisoning. Our patient presented with proteinuria after acetone ingestion and had AKI, which resolved by day five of admission. Her proteinuria also resolved. AKI was likely caused by decreased kidney perfusion secondary to hypotension and cardiopulmonary arrest because the fractional excretion of sodium (FeNa) was 0.6, suggestive of a pre-renal cause of AKI. However, acetone possibly contributed to the kidney dysfunction because she had proteinuria, which is not typical of pre-renal AKI.

Endocrine System

Acetone exposure has been associated with hyperglycemia and anion gap metabolic acidosis [7,17]. Acetone metabolism results in increased glucose production [22]. Our patient presented with hyperglycemia, which resolved within 48 hours, and anion gap metabolic acidosis, which resolved within 24 hours.

Management of acetone poisoning

Management of acetone poisoning is mainly supportive and begins with the assessment and stabilization of breathing, airway, and circulation. In addition, the patient's vital signs, including temperature, pulse, respiratory rate, and blood pressure, should be monitored. Due to the rapid acetone absorption after oral ingestion, gastric lavage is usually not indicated in most cases of isolated acetone ingestion. Still, it may be considered in cases of massive ingestion if the patient presents within one hour of ingestion. In addition, hemodialysis should be considered in patients with massive ingestion of acetone with persistent hemodynamic instability despite aggressive treatment with intravenous fluid and pressors. Although hemodialysis has been shown to increase the elimination of acetone and isopropanol substantially, it should only be considered in severe life-threatening poisonings and is rarely required [22,23].

## Conclusions

We presented a case that proved to be fatal following ingestion of a large dose of acetone. Mortality secondary to acetone is very rare. Our patient presented with cardiopulmonary arrest, hypotension, tachycardia, hyperglycemia, AKI, thrombocytopenia, and elevated liver enzymes; she also had extensive esophageal and gastric erosion with associated upper gastrointestinal bleeding. Management of acetone poisoning involves mainly supportive care.
